# Total and regional skeletal muscle mass measured by magnetic resonance imaging in Japanese children aged 7–11 years

**DOI:** 10.1038/s41598-026-45616-9

**Published:** 2026-03-28

**Authors:** Taishi Midorikawa, Megumi Ohta, Yuki Hikihara, Suguru Torii, Shizuo Sakamoto

**Affiliations:** 1 College of Health and Welfare, J.F. Oberlin University, 3758 Tokiwamachi, Machida, 194-0294 Tokyo Japan; 2 Waseda Institute for Sport Sciences, Waseda University, 2-579-15 Mikajima, Tokorozawa, 359-1192 Saitama Japan; 3 Faculty of Liberal Arts and Sciences, Chukyo University, 101-2 Yagoto Honmachi, Showa-ku, Nagoya-shi, 466-8666 Aichi Japan; 4 Faculty of Creative Engineering, Chiba Institute of Technology, 2-1-1 Shibazono, Narashino, 275-0023 Chiba Japan; 5 Faculty of Sport Sciences, Waseda University, 2-579-15 Mikajima, Tokorozawa, 359-1192 Saitama Japan

**Keywords:** Skeletal muscle mass, MRI, Children, Musculoskeletal system, Paediatric research

## Abstract

The aims of this study were to obtain representative data on total and regional skeletal muscle (SM) mass in prepubertal Japanese children using magnetic resonance imaging (MRI), and to examine sex differences in SM mass and its distribution. A total of 129 healthy prepubertal Japanese children (78 boys and 51 girls), aged 7–11 years and classified as Tanner stage 1, were enrolled. Contiguous MRI scans were acquired from the first cervical vertebra to the ankle joint. SM volume was calculated by summing digitised cross-sectional areas and dividing them into discrete anatomical regions. Age was significantly correlated with total, arm, trunk, thigh, and lower leg SM mass in both sexes. Total SM mass ranged from approximately 7 to 11 kg in boys and 6 to 10 kg in girls across the age range, increasing by roughly 1 kg per year in both groups. Boys had significantly higher intercepts in the regression lines between age and total, arm, trunk, and thigh SM mass compared to girls (*p* < 0.05), except for the lower leg. While no sex differences were observed in the slopes of the regression lines for total, arm, trunk, and lower leg SM mass, boys demonstrated a significantly steeper slope for thigh SM mass. This study provides detailed MRI-based reference representative data on SM mass and regional distribution in prepubertal children, contributing to a better understanding of early developmental differences between sexes.

## Introduction

Magnetic resonance imaging (MRI) has become the reference standard for measuring total and regional skeletal muscle (SM) mass, following validation against cadaver studies in the 1990s^1^. In a seminal study, Janssen et al. reported normative values for total body SM mass and its distribution in 468 healthy adults aged 18–88 years, using MRI^2^. Their findings indicated that sex differences in SM mass are more pronounced in the upper body, and that age-related declines in total body SM mass are primarily attributable to losses in the lower body, particularly after the fifth decade of life^2^. Standard reference values for SM mass are essential for understanding physiological characteristics across various disciplines, including nutrition, health, and sports sciences.

To date, MRI-based data on total body SM mass in children remain limited to a small number of pioneering studies. Hsu et al. investigated the relationship between SM mass (as a percentage of body weight) and resting energy expenditure in eight boys and seven girls aged 6–12 years^3^. Furthermore, MRI-derived total body SM mass has been employed to develop paediatric prediction equations using dual-energy X-ray absorptiometry (DXA) in 46 boys and 37 girls aged 5–14 years^4^, and whole-body potassium-40 (^40^K) counting in 66 males and 50 females aged 5–17 years^5^. However, large-scale studies reporting regional SM mass in children using MRI remain scarce.

Accurate assessment of SM mass in paediatric populations requires high-resolution imaging. Previous studies have typically acquired approximately 30–40 cross-sectional MRI slices to capture the whole body, often using slice intervals of 25–35 mm, or up to 40 mm^3–5^. Such approaches may limit the precision of regional SM quantification. Therefore, the aims of the present study were to obtain representative data on total and regional SM mass in a cohort of over 100 prepubertal Japanese boys and girls using contiguous whole-body MRI scans with no interslice gap, and to examine sex differences in SM mass and its distribution.

## Methods

### Subjects

**Fig. 1:**
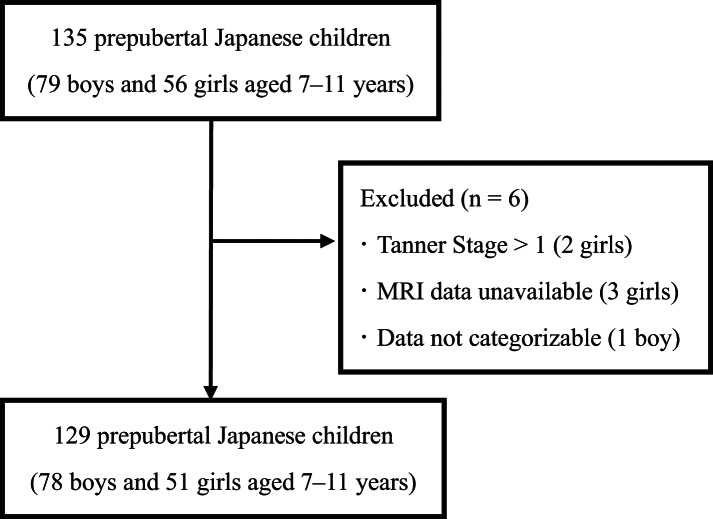
Flowchart illustrating the study sample selection.

A total of 129 healthy prepubertal Japanese children (78 boys and 51 girls), aged 7–11 years and classified as Tanner Stage 1, were finally enrolled for this study through referrals from acquaintances and personal contacts in Tokyo. Data collection was conducted between August 2007 and August 2010 (Fig. 1). Maturation status was assessed by trained research staff using the Tanner scale questionnaire^6^. All participants were physically active, regularly engaging in outdoor play; however, none were involved in organised sports or athletic training. None of the children had any known pathological conditions or were taking medication at the time of the study. The research protocol adhered to the ethical standards outlined in the Declaration of Helsinki and was approved by the Ethics Review Committee on Research with Human Subjects of Waseda University at the time of data collection. The study was conducted as a continuing research project across multiple academic years. Written informed consent was obtained from all participants and their parents or legal guardians prior to inclusion in the study.

### Anthropometric measurements

Standing height was measured to the nearest 0.1 cm using a stadiometer (YS-OA, AS ONE Corporation, Osaka, Japan), and body weight was measured to the nearest 0.1 kg using a digital balance (DC-320, TANITA Corporation, Tokyo, Japan), with participants dressed in minimal clothing. Body mass index (BMI) was calculated as body weight in kilogrammes divided by the square of height in metres (kg/m²). Body fat percentage was assessed using DXA (Delphi A-QDR, Hologic Inc., Marlborough, MA, USA) with the paediatric whole-body software (Version 12.4:3). Fat-free mass (FFM) was derived by subtracting fat mass from total body weight.

### Skeletal muscle mass measurement by MRI

Total body SM volume was measured using a 1.5-Tesla MRI scanner (Signa EXCITE VI, GE Healthcare, Chicago, IL, USA). A T1-weighted spin-echo sequence was employed in the axial plane, with a repetition time of 500 ms and an echo time of 13.1 ms. Depending on the imaging region, data were acquired during either breath-holding or normal breathing. Participants lay supine in the scanner with their hands placed on the abdomen. Contiguous transverse images with a slice thickness of 1.0 cm and no interslice gap were acquired from the first cervical vertebra to the lateral malleolus. Approximately five sets of images from the first cervical vertebra to the femoral head were acquired during breath-holding (each lasting approximately 20 s), while the remaining sets from the femoral head to the ankle joints were obtained during normal breathing^7^.

Each image set comprised approximately 100–150 slices per subject. These were manually segmented by a team of highly trained technicians to isolate SM tissue, excluding connective tissue, blood vessels, fat, and abdominal organs. Cross-sectional areas of skeletal muscle were analysed using ZedView software (LEXI Co., Ltd., Tokyo, Japan), and total SM volume was calculated by summing the areas (cm²) across all slices and multiplying by the slice thickness (cm). The resulting volume (cm^3^) was converted to mass (kg) using a muscle density of 1.041 g/cm^8^. The test–retest coefficient of variation for SM volume measurement was estimated at 2%^7^. Total SM mass was further divided into anatomical regions based on distinct imaging landmarks: the arm (from the axillary fossa to the styloid process of the radius), the trunk (from the first cervical vertebra to the femoral neck), the thigh (from the femoral neck to the articular surface of the medial condyle), and the lower leg (from the articular surface of the medial condyle to the lateral malleolus).

### Statistical analysis

All data are presented as mean ± standard deviation (SD). Normality of distribution was assessed using the Shapiro–Wilk test. Relationships between age, standing height, and total or regional SM mass were evaluated using both Pearson product-moment and Spearman rank correlation coefficients, as appropriate. Sex differences in the regression slopes and intercepts for SM mass variables were assessed using analysis of covariance (ANCOVA). All statistical analyses were performed using SPSS for Windows (version 29.0; SPSS Inc., Armonk, NY, USA). Additionally, a piecewise regression model was constructed to examine the relationship between standing height and total SM mass, using R software (version 4.3.3; R Core Team, R Foundation for Statistical Computing, Vienna, Austria) in RStudio (version 2024.04.1 + 748; Chocolate Cosmos, Posit Software, PBC, USA).

## Results

**Fig. 2:**
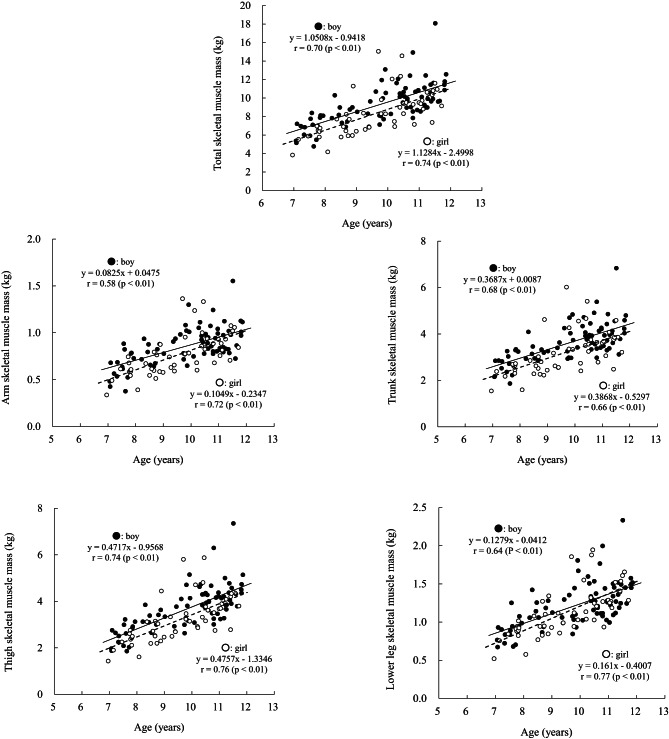
Relationship between age and total and regional skeletal muscle mass. Solid line: boys; dashed line: girls.

The physical characteristics and the absolute and relative values of SM mass are summarised in Tables 1 and 2. Age was significantly correlated with total, arm, trunk, thigh, and lower leg SM mass in both boys and girls (Fig. 2). No significant sex differences were observed in the slopes of the regression lines between age and SM mass (Fig. 2). However, boys had significantly higher intercepts in the regression lines for total, arm, trunk, and thigh SM mass compared with girls (*p* < 0.05), except for the lower leg.

**Table 1:** Subject characteristics.

years	*n*	Standing height (m)			Body weight (kg)			BMI (kg/m^2^)			Fat (%)			Fat free mass (kg)		
Boys																
7	15	1.24	±	0.06	25.6	±	5.9	16.6	±	2.6	23.7	±	7.8	19.2	±	2.5
8	9	1.31	±	0.04	31.2	±	7.2	18.0	±	3.3	26.6	±	8.6	22.4	±	2.6
9	12	1.35	±	0.08	37.0	±	10.3	19.4	±	3.6	30.0	±	9.4	25.0	±	4.0
10	22	1.41	±	0.06	34.2	±	6.4	17.2	±	2.0	21.7	±	7.1	26.4	±	3.3
11	20	1.45	±	0.07	36.3	±	9.4	17.1	±	3.1	22.7	±	7.5	27.5	±	4.4
all	78	1.37	±	0.10	33.2	±	8.7	17.5	±	2.9	24.2	±	8.2	24.6	±	4.6
Girls																
7	10	1.24	±	0.05	25.3	±	4.4	16.5	±	3.1	28.0	±	5.8	18.0	±	2.2
8	11	1.28	±	0.07	28.1	±	7.0	16.9	±	2.9	29.9	±	7.3	19.4	±	4.1
9	8	1.34	±	0.09	31.9	±	9.7	17.4	±	3.3	27.4	±	8.8	22.8	±	6.2
10	12	1.44	±	0.06	37.4	±	9.0	17.9	±	3.0	29.5	±	8.3	26.0	±	5.1
11	10	1.45	±	0.04	37.5	±	6.5	17.9	±	2.9	26.2	±	6.2	27.4	±	3.5
all	51	1.35	±	0.10	32.2	±	8.8	17.3	±	3.0	28.3	±	7.2	22.8	±	5.6

n, number of subjects. BMI, body mass index.

**Table 2:** Absolute and relative values of skeletal muscle mass.

years		SMM (kg)															Total SMM/ standing height (m)			Total SMM/ body weight (kg)			Total SMM/ fat free mass (kg)		
	*n*	Total body			Arm			Trunk			Thigh			Lower leg											
Boys																									
7	15	6.74	±	1.20	0.63	±	0.15	2.71	±	0.48	2.51	±	0.47	0.88	±	0.16	5.42	±	0.76	0.27	±	0.03	0.35	±	0.02
8	9	8.30	±	1.07	0.78	±	0.11	3.20	±	0.40	3.19	±	0.43	1.13	±	0.17	6.30	±	0.65	0.27	±	0.03	0.37	±	0.01
9	12	9.47	±	1.83	0.91	±	0.18	3.67	±	0.66	3.67	±	0.76	1.23	±	0.29	6.97	±	1.07	0.26	±	0.04	0.38	±	0.02
10	22	10.30	±	1.58	0.93	±	0.12	3.98	±	0.57	4.08	±	0.70	1.32	±	0.24	7.30	±	0.84	0.30	±	0.03	0.39	±	0.02
11	20	10.83	±	2.07	0.95	±	0.19	4.13	±	0.80	4.35	±	0.87	1.39	±	0.28	7.43	±	1.10	0.30	±	0.03	0.39	±	0.02
all	78	9.39	±	2.21	0.86	±	0.19	3.64	±	0.81	3.68	±	0.96	1.22	±	0.30	6.81	±	1.17	0.29	±	0.04	0.38	±	0.02
Girls																									
7	10	6.13	±	0.95	0.58	±	0.11	2.42	±	0.36	2.28	±	0.39	0.85	±	0.14	4.95	±	0.81	0.24	±	0.02	0.34	±	0.02
8	11	6.96	±	1.82	0.63	±	0.14	2.66	±	0.76	2.70	±	0.74	0.97	±	0.22	5.38	±	1.14	0.25	±	0.02	0.35	±	0.02
9	8	8.77	±	2.75	0.81	±	0.25	3.49	±	1.17	3.36	±	1.05	1.11	±	0.31	6.45	±	1.49	0.28	±	0.03	0.38	±	0.01
10	12	9.63	±	2.30	0.90	±	0.22	3.66	±	0.87	3.75	±	0.94	1.32	±	0.32	6.65	±	1.31	0.26	±	0.03	0.37	±	0.02
11	10	9.88	±	1.25	0.91	±	0.13	3.63	±	0.58	3.93	±	0.48	1.41	±	0.16	6.81	±	0.74	0.27	±	0.03	0.36	±	0.02
all	51	8.28	±	2.37	0.77	±	0.22	3.17	±	0.92	3.21	±	0.96	1.14	±	0.32	6.04	±	1.31	0.26	±	0.03	0.36	±	0.02

n, number of subjects. SMM, skeletal muscle mass.

**Fig. 3:**
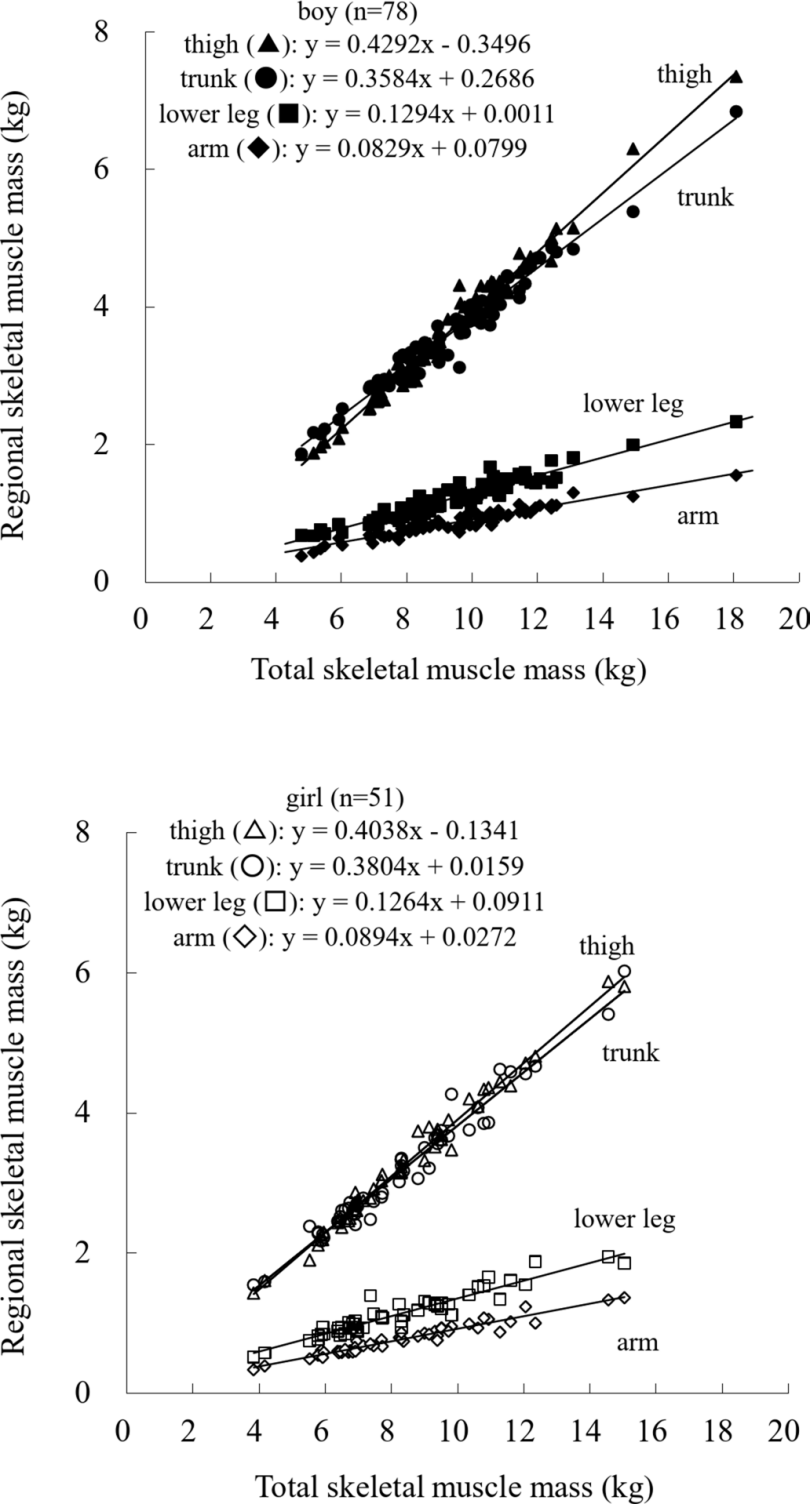
Relationship between total and regional skeletal muscle masses. All regional SM values were significantly correlated with the total SM (*p* < 0.01).

Regression analyses revealed significant correlations between total and all regional SM masses in both sexes (Fig. 3). The slope of the regression line for thigh SM mass relative to total SM mass was approximately 0.43 kg/kg in boys and 0.40 kg/kg in girls, which was greater than the corresponding slopes for the other regions (Fig. 3). While no significant sex differences were observed in the slopes for the arm, trunk, or lower leg, boys exhibited a significantly steeper slope for thigh SM mass relative to total SM mass than girls (*p* < 0.05).

**Fig. 4:**
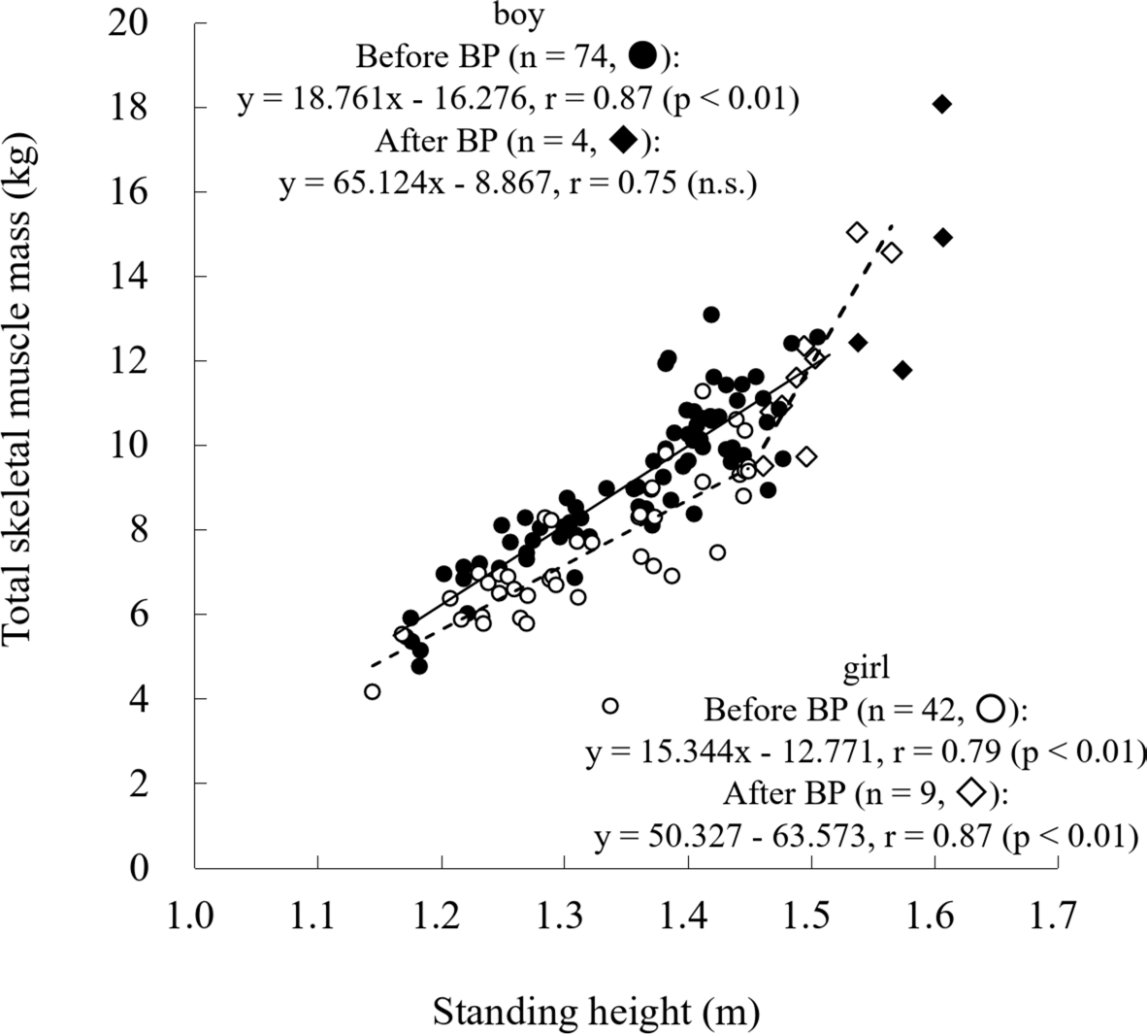
Relationship between standing height and total skeletal muscle mass. Solid line: boys; dashed line: girls. BP, breakpoint.

Piecewise regression analysis of the relationship between standing height and total SM mass identified breakpoints at 1.51 m for boys and 1.45 m for girls. In girls, the slope increased from 15.34 to 50.33 before and after the breakpoint, respectively. In contrast, no significant correlation was observed after the breakpoint in boys, likely due to the small sample size beyond this point (*n* = 4) (Fig. 4).

## Discussion

While previous cadaver studies provided limited information on children’s skeletal muscle (SM) mass^9,10^, the total SM mass observed in the present study ranged from approximately 7 to 11 kg in boys and 6 to 10 kg in girls between the ages of 7 and 11, increasing by approximately 1 kg per year in both sexes (Table 2; Fig. 2). The mean standing height and body weight of participants were comparable to the values reported in the *Physical Fitness Standards of Japanese People* (Tokyo Metropolitan University Press)^11^(z-score = 0.34 ± 1.08 for boys and 0.31 ± 1.04 for girls). Although the prevalence of childhood obesity (defined as ≥ 20% above the standard body weight) was slightly higher in this study (14% for boys and 18% for girls) than the approximately 10% reported for both sexes in the *School Health Statistics Survey* in Japan after 2006, the total SM mass and its distribution observed here, based on relatively large-scale and detailed MRI analysis, can be considered representative of prepubertal Japanese children.

The ratio of total body SM mass to standing height increased by approximately two units in both boys and girls between the ages of 7 and 11, whereas the ratios of total SM mass to body weight and fat-free mass (FFM) showed little change (Table 2). A previous study reported that ultrasound-derived SM prediction equations, based on muscle thickness and standing height in adults, were applicable to adolescents with near-adult SM-to-height ratios (12.1 kg/m for boys and 9.4 kg/m for girls)^12^. As standing height contributes to the length component of SM development, the ratio of total SM mass to height may serve as a useful index of skeletal muscle maturation during childhood.

The present study also demonstrated that the SM-to-height ratio increased at estimated breakpoints of 1.51 m for boys and 1.45 m for girls (Fig. 4). These values are comparable to those reported in a previous ultrasonography-based study of 271 Japanese children aged 6–19 years, which showed that limb SM cross-sectional area increased rapidly, reaching approximately 1.53 m for boys and 1.40 m for girls^13^. These similarities support the validity of the current findings. However, further research involving a larger number of participants beyond the breakpoint (i.e., four boys and nine girls in the present study) is needed to clarify whether the shape and trajectory of SM development differ before and after this transition.

Despite the importance of understanding SM distribution in growth, few studies have compared SM distribution between children and adults. A previous MRI study of young Japanese adults, which used the same anatomical landmarks as the present study^7^, reported that the proportion of total SM mass was 43% and 41% in the trunk, 36% and 37% in the thigh, 11% and 13% in the lower leg, and 10% and 9% in the arms for males and females, respectively. In comparison, the current data for prepubertal children revealed a slightly lower proportion of trunk SM and a higher proportion in the thigh and lower leg (39% and 38% in the trunk, 39% and 39% in the thigh, 13% and 14% in the lower leg, and 9% and 9% in the arms for boys and girls, respectively, at an average age of 9 years). Although longitudinal studies are needed to confirm developmental changes, these findings suggest that prepubertal children may possess a relatively greater proportion of SM in the lower body compared with adults.

Only a few studies have established SM reference values for prepubertal children using methods such as segmental bioelectrical impedance^14^ and DXA^15^. Consistent with previous research, boys in this study exhibited higher total SM mass than girls, as indicated by the regression line intercepts. A novel finding of this study is that boys also had greater arm, trunk, and thigh SM mass than girls, whereas no significant sex difference was observed in lower leg SM mass. The absence of difference in the lower leg remains unclear. Additionally, the steeper slope between total and thigh SM mass observed in boys suggests that sex-related differences in thigh muscle development may begin to emerge even during prepubertal years.

Information on total and regional SM mass in prepubertal children is of considerable relevance to paediatrics, nutrition, child health, and sports sciences. To our knowledge, no previous study has conducted such a detailed MRI-based assessment of SM in prepubertal children. Therefore, this study provides a valuable and representative dataset. Nevertheless, due to the relatively small sample size within specific age groups, further data collection is warranted to enhance the reliability and generalisability of these findings.

## Data Availability

The datasets generated and/or analysed during the current study are not publicly available due to restrictions, for example, they contain information that could compromise the privacy of research participants, but are available from the corresponding author upon reasonable request.

## Abbreviations

BMI: body mass index
DXA: dual-energy X-ray absorptiometry
FFM: fat-free mass
MRI: magnetic resonance imaging
SM: skeletal muscle
